# Successful ablation of lower loop re-entry after Starnes procedure in an infant with severe tricuspid valve dysplasia: a case report

**DOI:** 10.1093/ehjcr/ytag777

**Published:** 2026-10-24

**Authors:** Ken Hayashi, Hisaaki Aoki, Yoichiro Ishii, Kazuki Tanimoto, Sanae Tsumura

**Affiliations:** Department of Cardiology, Osaka Women’s and Children’s Hospital, 840 Murodocho, Izumi, Osaka 594-1101, Japan; Department of Cardiology, Osaka Women’s and Children’s Hospital, 840 Murodocho, Izumi, Osaka 594-1101, Japan; Department of Cardiology, Osaka Women’s and Children’s Hospital, 840 Murodocho, Izumi, Osaka 594-1101, Japan; Department of Cardiovascular Surgery, Osaka Women’s and Children’s Hospital, 840 Murodocho, Izumi, Osaka 594-1101, Japan; Department of Cardiovascular Surgery, Osaka Women’s and Children’s Hospital, 840 Murodocho, Izumi, Osaka 594-1101, Japan

**Keywords:** Atypical flutter, Lower loop re-entry, Infant, Ablation, Tricuspid valve dysplasia, Starnes procedure, Case report

## Abstract

**Background:**

Tricuspid valve dysplasia (TVD) is a rare right-sided congenital heart disease characterized by severe tricuspid regurgitation and right atrial enlargement, which may predispose to atrial arrhythmias. In severe neonatal cases, the Starnes procedure can be life-saving, but postoperative anatomical alterations may complicate subsequent arrhythmia management.

**Case summary:**

A 2-month-old female infant with TVD and pulmonary atresia developed drug-refractory atypical atrial flutter after the Starnes procedure. Three-dimensional electroanatomical mapping identified lower loop re-entry involving the cavotricuspid isthmus (CTI). Radiofrequency catheter ablation across the CTI successfully terminated the tachycardia. However, atypical flutter recurred 1 week later. During subsequent systemic pulmonary shunt surgery, additional surgical ablation was performed using the prior catheter ablation site as an anatomical landmark. After detaching the tricuspid valve patch, the CTI line was extended to the tricuspid annulus, resulting in complete arrhythmia control.

**Discussion:**

The outcome of this case suggests that foetal-onset volume overload from severe tricuspid regurgitation may promote early atrial remodelling and arrhythmogenic substrate formation in TVD. Although CTI ablation after the Starnes procedure may be feasible in a small infant, patch-related anatomic barriers may prevent complete lesion formation. Prior catheter ablation sites may be useful intraoperative landmarks for successful surgical ablation.

Learning points

- Foetal-onset severe tricuspid regurgitation imposes chronic volume overload on the right atrium, leading to early myocardial remodelling, which may serve as a substrate for arrhythmogenesis.
- After the Starnes procedure, access to the cavotricuspid isthmus is hindered by the tricuspid valve patch, presenting challenges in radiofrequency catheter ablation.
- Even if atrial tachycardia recurs after radiofrequency catheter ablation, subsequent surgical ablation can be effectively guided using the radiofrequency catheter ablation lesion site as an anatomical landmark.

## Introduction

Tricuspid valve dysplasia (TVD) is a right-sided congenital heart disease that shares features with Ebstein anomaly, particularly the presence of significant tricuspid regurgitation, leading to enlargement of the right atrium and ventricle. In severe cases, cardiomegaly compresses the lungs, causing postnatal respiratory and circulatory failure, necessitating an early neonatal Starnes procedure. The enlarged right atrium and surgical incision lines can serve as arrhythmogenic substrates. In the present case, we performed catheter ablation using a 3D mapping system for drug-refractory tachycardia in an infant weighing 3.2 kg, after the Starnes procedure. Mapping identified lower loop re-entry, which was terminated using radiofrequency catheter ablation (RFA) along the cavotricuspid isthmus (CTI) line. For subsequent atypical flutter recurrence, during systemic pulmonary shunt surgery, the previous catheter ablation site was used as an anatomical landmark to guide additional surgical ablation, which successfully terminated the arrhythmia.

## Summary figure

**Figure:**
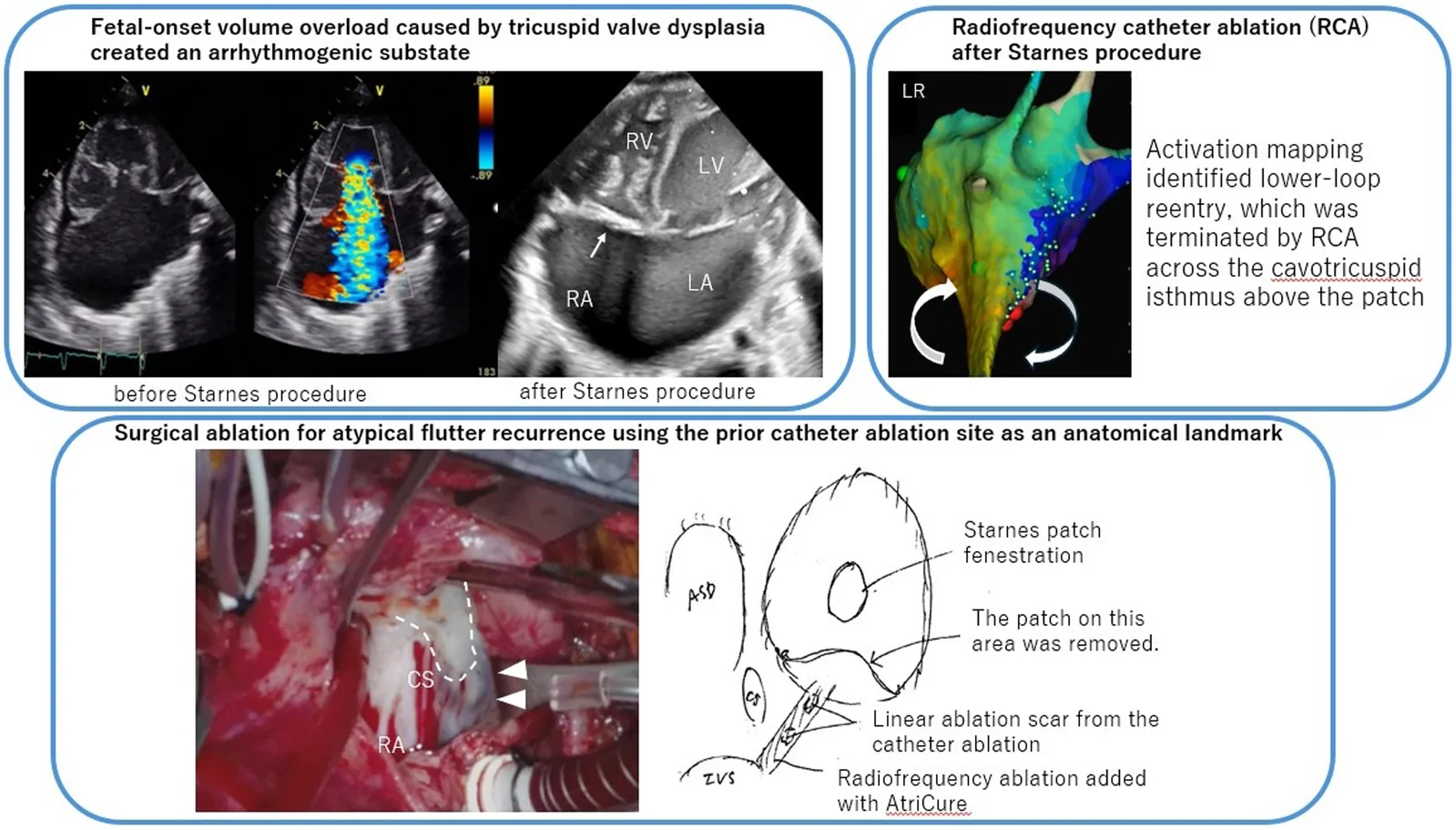


## Case presentation

The patient was a 2-month-old female infant with both TVD and atypical flutter. During the foetal period, she was diagnosed with TVD and pulmonary atresia accompanied by severe tricuspid regurgitation and marked right atrial enlargement. Upon detection of foetal heart failure, she was delivered via emergency caesarean section at 37 weeks and 3 days of gestation, with a birth weight of 2258 g. Immediately after birth, she was intubated and managed with 100% fraction of inspired oxygen, inhaled nitric oxide, and a catecholamine infusion. With the patient in the prone position, oxygen saturation was maintained at 70%–80%. On Day 1 after birth, the Starnes procedure was performed, comprising patch closure of the tricuspid valve with a 4-mm fenestration, right atrial plication, and atrial septostomy, together with bilateral pulmonary artery banding while maintaining ductal patency (*Figure 1B*). Occurrence of atypical flutter began on postoperative Day 2. Atrial overdrive pacing and synchronized cardioversion were attempted, but tachycardia recurred immediately. Pharmacological cardioversion using landiolol, flecainide, amiodarone, and nifekalant was unsuccessful. The ventricular rate was controlled at 150–200 bpm using landiolol and amiodarone. Based on the echocardiography and contrast-enhanced computed tomography findings, the patch was sutured to the tricuspid annulus. Therefore, we considered that linear ablation would be feasible even if the atypical flutter represented a CTI-dependent macroreentrant circuit (*Figure 1C* and *D*). Catheter ablation was planned at the time of cardiac catheterization, before systemic pulmonary shunt surgery, because the mechanism underlying the supraventricular tachycardia remained unclear and successful ablation could have prevented the need for a right atriotomy during the planned shunt procedure.

**Figure 1:**
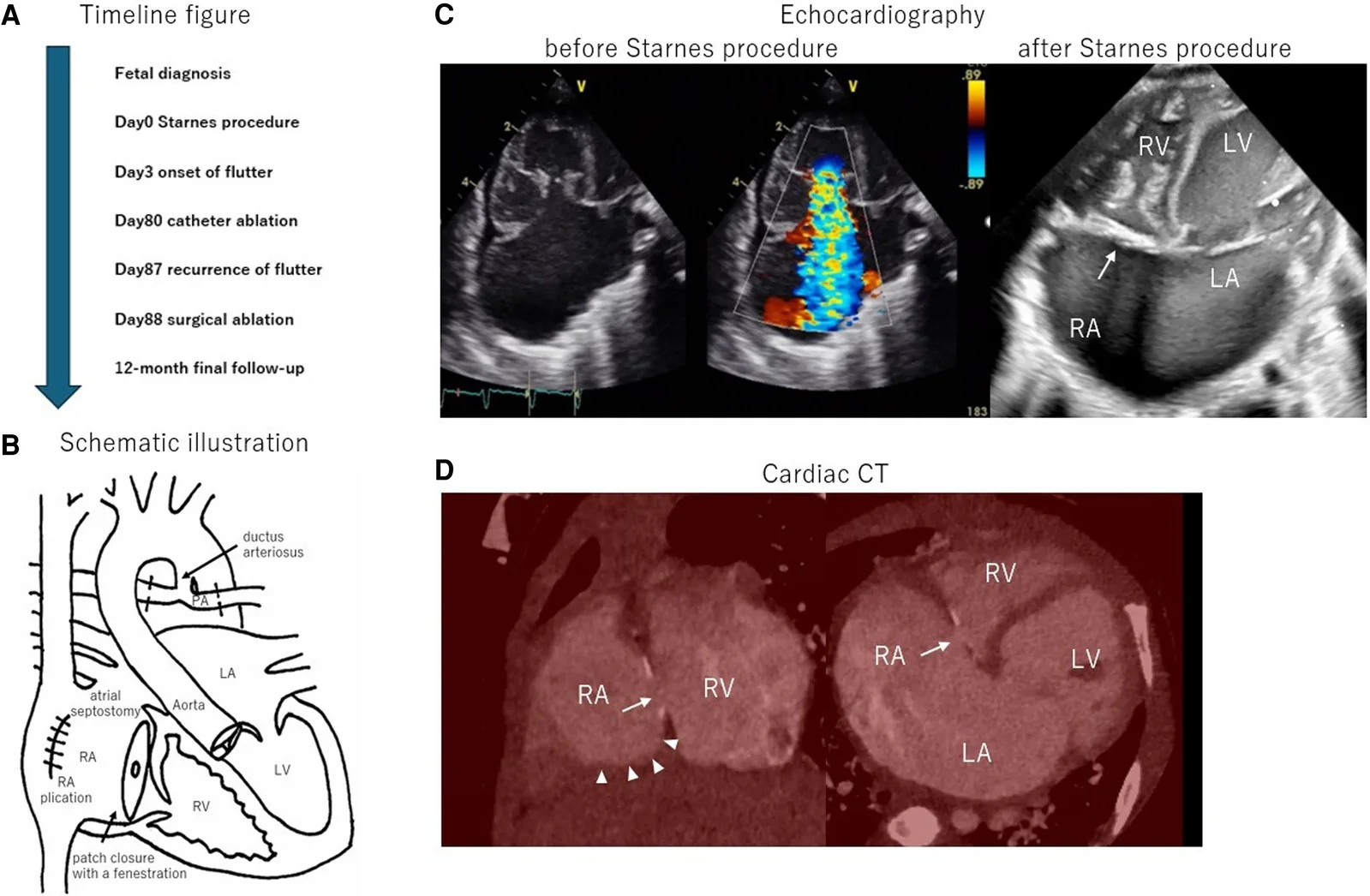
(*A*) Timeline of the patient’s clinical course and treatment. (*B*) Schematic illustration of the patient’s heart after the Starnes procedure. (*C*) Echocardiography before and after the Starnes procedure. Severe tricuspid regurgitation occurred due to tricuspid dysplasia was found (left). Gore-Tex patch was sutured along the tricuspid annulus (arrow). (*D*) Cardiac computed tomography revealed a Starnes patch along the tricuspid annulus and the cavotricuspid isthmus, which can be completely ablated with linear ablation. RA, right atrium, RV, right ventricle, LA, left atrium, LV, left ventricle.

When the infant was 2 months and 21 days old and weighed 3.2 kg, we performed RFA of the atypical flutter under general anaesthesia, using an EnSite X™ Mapping System (Abbott Laboratories, Abbott Park, IL, USA). Two-electrode catheters were positioned in the right atrium and at the His-bundle region using a 5-Fr guiding sheath (MPX; Japan Lifeline Co., Tokyo, Japan) from the left femoral vein. A 6-Fr (2 mm) bipolar pacing oesophageal catheter (FIAB Esokids 4S; Firenze, Italy) was positioned in the oesophagus. At the beginning of the intervention, the atypical flutter was sustained. The P-wave morphology was flat in lead I, positive in leads II, III, and aVF, and positive in V1 (*Figure 2A*). After constructing an activation map, entrainment pacing was performed in the atrium. The post-pacing interval minus tachycardia cycle length (PPI−TCL) was 67 ms in the left atrium, 46 ms in the high right atrium, 5 ms in the lower posterior right atrium, and 0 ms at the CTI (*Figure 2D*). Based on the activation map and entrainment findings, the atypical flutter was diagnosed as a lower loop re-entry (*Figure 2B*, Supplemental Material 1). During atypical flutter, fragmented potentials were observed in the CTI (*Figure 3A*). Linear ablation across the CTI was performed at eight sites during tachycardia using a 4-mm tip steerable catheter (Ablaze Fantasista, 5Fr, Japan Lifeline, Tokyo, Japan) in temperature control mode (maximum 70°C, 30 W) (*Figure 4B*). Regarding the atrioventricular node, the ablation site was sufficiently distant from the atrioventricular node. In addition, radiofrequency energy was delivered while continuously confirming atrioventricular conduction, further enhancing procedural safety. RFA terminated the tachycardia, and no recurrence was observed during a 30-min observation period. Following ablation, the Wenckebach point was 210 beats/min, indicating preserved AV nodal conduction. Atrial burst pacing was performed up to 350 beats/min, but no arrhythmia was induced. Bidirectional conduction block across the ablation line was confirmed. The primary objectives of the electrophysiological study and ablation in the present case were to diagnose the atrial tachycardia and identify its re-entrant circuit. We had anticipated that, if catheter ablation was unsuccessful, additional ablation could be performed during subsequent cardiac surgery. After percutaneous ablation, landiolol and amiodarone were discontinued. However, the arrhythmia recurred 1 week after the procedure, and landiolol was restarted. During shunt creation, additional surgical ablation was performed using an AtriCure bipolar radiofrequency ablation device (AtriCure, Inc., Mason, OH, USA), targeting the area corresponding to the previous catheter ablation site, which appeared to be darkened due to prior radiofrequency energy delivery (*Figure 4*, Supplemental Material 2). The CTI was identified based on the anatomical relationship between the vessels and the heart, as well as changes in tissue colouration. The recurrence may have been attributable to inadequate lesion formation despite the apparent completion of the ablation line. Alternatively, a complete CTI line may not have been established at the end of the initial procedure because the presence of the patch prevented the ablation line from being extended to the tricuspid annulus. Accordingly, during the surgical procedure, we performed ablation along the entire CTI. After detaching the patch, we extended the previously incomplete CTI line to the level of the tricuspid annulus. The intraoperatively resected atrial tissue presented with severe myocardial injury (*Figure 5*). At the final follow-up, 9 months after ablation, sinus rhythm was seen, and no premature atrial contractions, premature ventricular contractions, or supraventricular tachycardia were observed on Holter electrocardiography.

**Figure 2:**
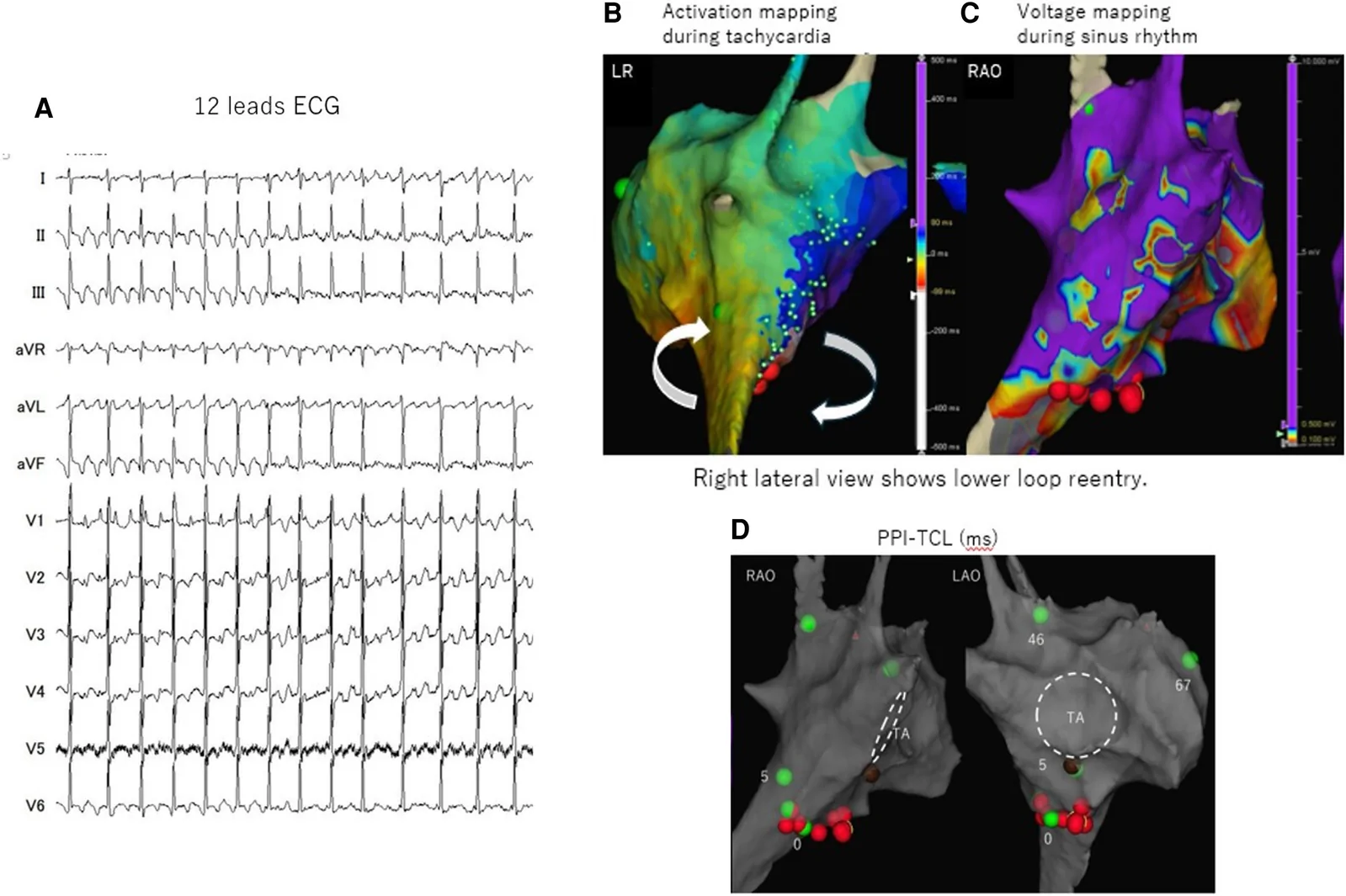
(*A*) Twelve-lead electrocardiography showing atypical flutter with changes in P wave morphology and P rate. (*B*) Activation mapping during tachycardia (atrial cycle length 174 ms) showing lower loop re-entry. (*C*) Voltage mapping during sinus rhythm. (*D*) The post-pacing interval minus tachycardia cycle length (PPI−TCL) was 67 ms in the LA, 46 ms in the high RA, 5 ms in the posterior lower RA, and 0 ms at the cavotricuspid isthmus. PPI, post-pacing interval; TCL, tachycardia cycle length; LA, left atrium; RA, right atrium.

**Figure 3:**
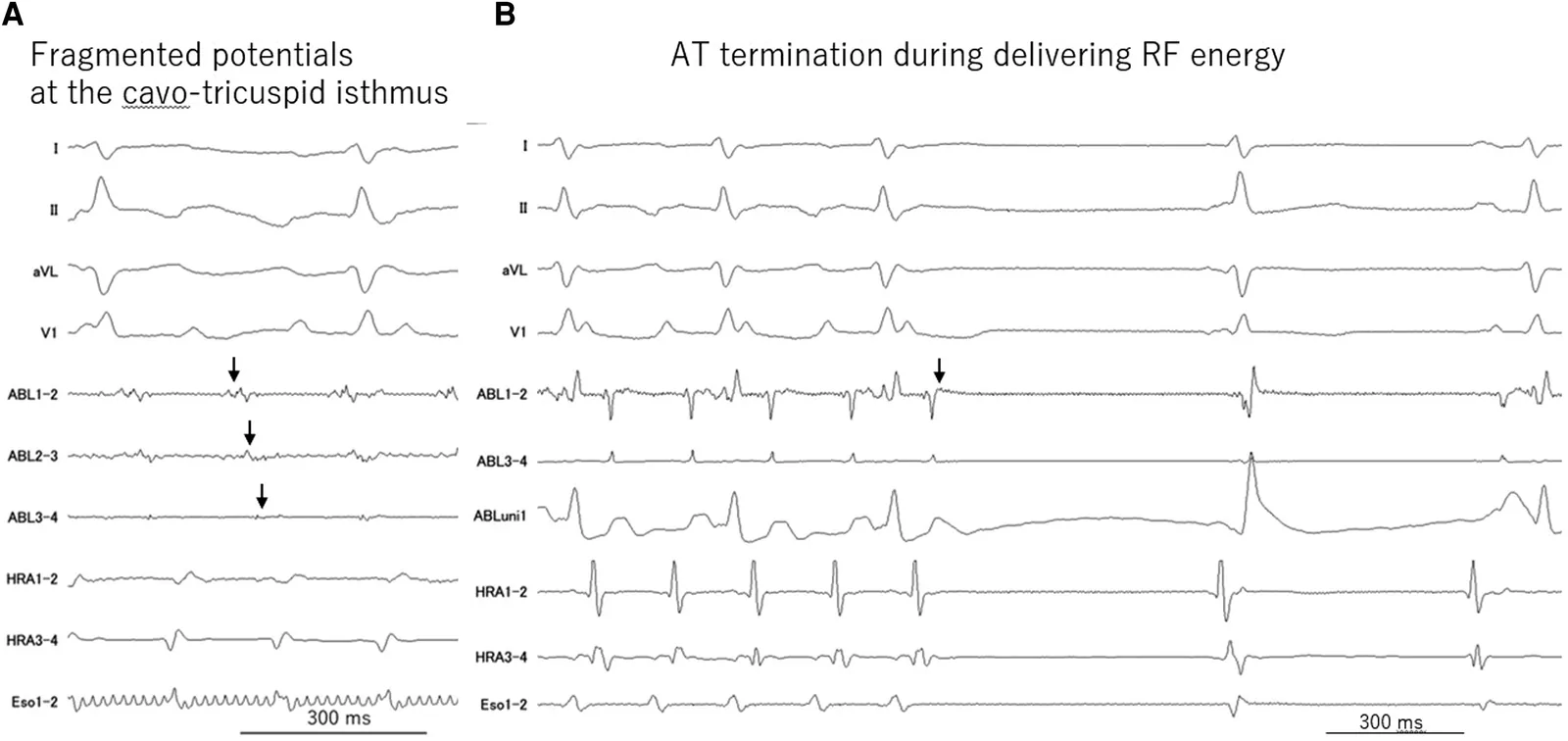
(*A*) Fragmented potentials were recorded at the cavotricuspid isthmus during the tachycardia. The arrows indicate fragmented potentials. (*B*) Atypical flutter terminated by delivering RF energy during tachycardia. The arrow indicates block of the tachycardia circuit. RF, radiofrequency.

**Figure 4:**
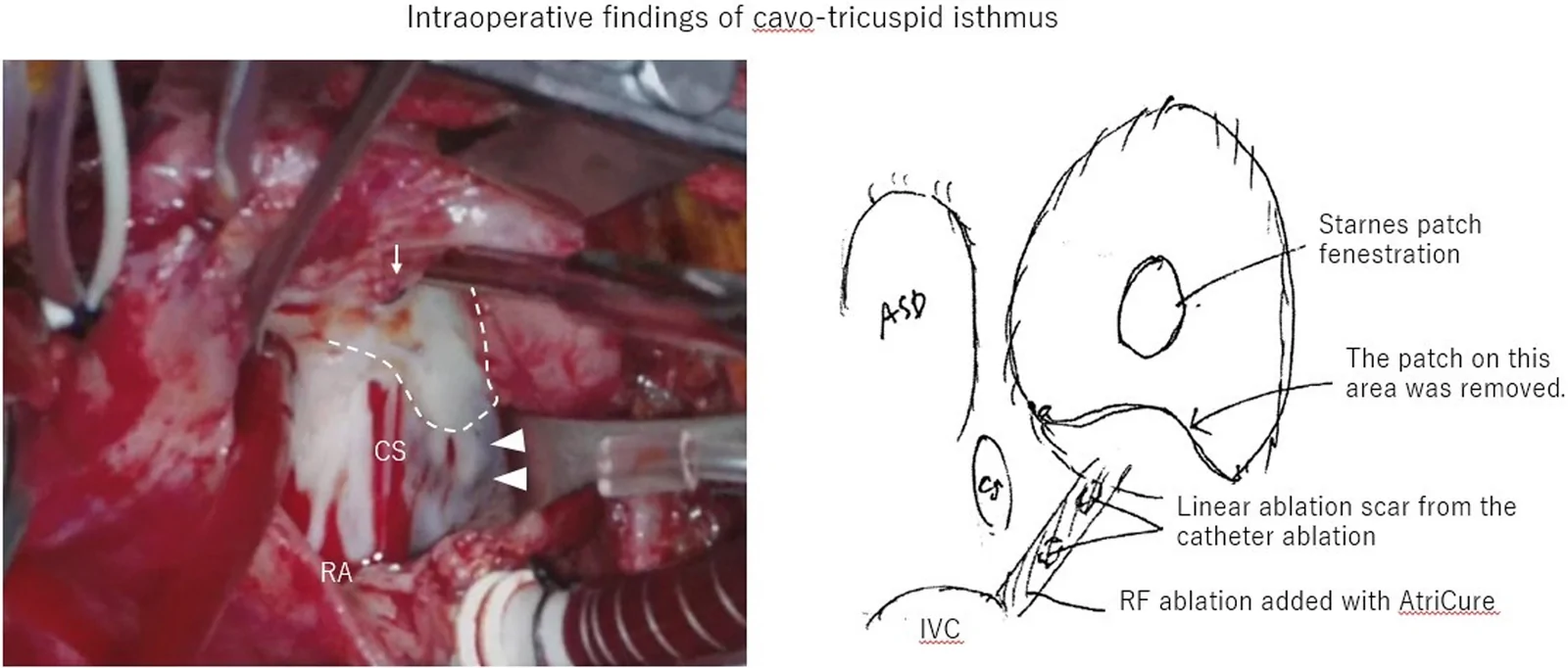
Intraoperative findings. The scar of the previous catheter ablation was recognized at the cavotricuspid isthmus (arrowheads). Additional surgical ablation was performed using an AtriCure bipolar radiofrequency ablation device (AtriCure, Inc., Mason, OH, USA), targeting the area corresponding to the previous catheter ablation site as an anatomical landmark (supplement). CS, coronary sinus ostium; RA, right atrium; dotted line, suture line between the tricuspid annulus and Starnes patch; arrow, fenestration of the Starnes patch.

**Figure 5:**
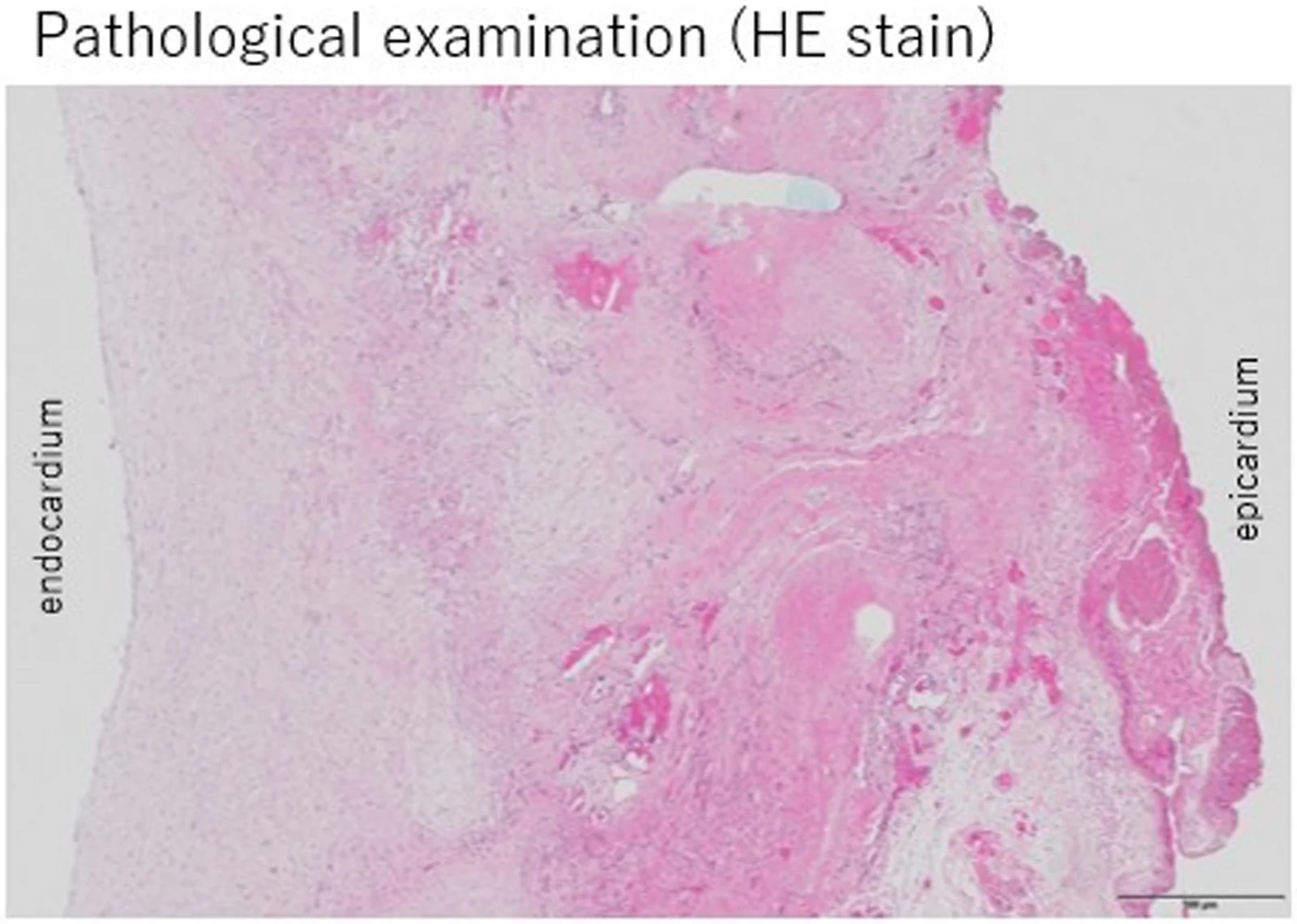
Pathological examination of excised atrial tissue showing marked endocardial thickening, degeneration in myocardial cells, interstitial fibrosis, and calcification are observed (haematoxylin and eosin staining).

## Discussion

This case report highlights two important findings. First, severe tricuspid regurgitation was present from the foetal period, resulting in foetal-onset volume overload, leading to early atrial myocardial remodelling and the formation of an arrhythmogenic substrate. Second, CTI ablation was feasible in a low-birthweight infant after the Starnes procedure. Furthermore, in the setting of postoperative tachycardia recurrence, the prior RFA lesion served as an anatomical landmark to guide subsequent surgical ablation.

We speculate that the refractory atypical atrial flutter in this case was associated with slow conduction caused by atrial myocardial damage secondary to longstanding severe tricuspid regurgitation beginning in the foetal period; though it is a limitation of this single-case report that foetal atrial remodelling alone cannot be conclusively declared as the underlying mechanism of the arrhythmia. Ebstein anomaly is a distinct disease entity with an arrhythmogenic substrate different from that of TVD, but it imposes chronic volume overload on the right atrium in a similar manner, leading to right atrial dilation and creating a fertile substrate for atrial arrhythmias.^1^ The most common acquired atrial arrhythmias related to Ebstein anomaly are macroreentrant circuits, including classical CTI-dependent flutter around the tricuspid valve annulus. Cardiac magnetic resonance imaging in cases of Ebstein anomaly has demonstrated right atrial fibrosis, which is significantly associated with supraventricular arrhythmias.^2^ The right atrium is intrinsically characterized by relatively sparse myocardial cells and abundant extracellular matrix; accordingly, it has been suggested that volume overload further promotes the development of a fibrotic substrate. In patients with TVD, right atrial enlargement has also been reported to serve as an arrhythmogenic substrate.^3,4^ In the present case, histopathological findings revealed myocardial cell degeneration and interstitial fibrosis, suggesting arrhythmogenic potential (*Figure 5*).

Catheter ablation is generally deferred in younger children, particularly those weighing less than 15 kg or younger than 4 years, because of vascular access limitations and higher complication rates; however, it may be considered for selected infants and small children who have drug-refractory or life-threatening tachyarrhythmias.^5^ In patients with congenital heart disease, the arrhythmogenic substrate is often complex,^6^ and postsurgical alterations in cardiac anatomy may render catheter access to the target site challenging.^7^ To the best of our knowledge, there have been no previous reports of CTI linear ablation for atypical flutter following the Starnes procedure. In this case, the tricuspid valve patch from the Starnes procedure rendered CTI access technically challenging. The recurrence of atypical flutter in the present case may have been due to insufficient ablation of the CTI segment extending from below the patch to the tricuspid annulus. Whether CTI linear ablation is feasible requires careful anatomical evaluation using imaging modalities like echocardiography and computed tomography. In patients with Ebstein anomaly, the success rate of CTI ablation after tricuspid valve repair or replacement is low, and the procedure is technically demanding, prompting recommendations for preoperative ablation.^8,9^ However, in young infants with a small body size, the atrial wall is thin, and the tricuspid valve tissue is fragile, raising concerns that prior catheter ablation may injure the valve apparatus and complicate subsequent surgical delamination or cone reconstruction.^10^ In such cases, right atrial cryosurgical maze procedures under direct vision have been proposed.

## Conclusion

3D mapping-guided catheter ablation effectively terminated lower loop re-entrant atypical flutter in an infant with TVD following the Starnes procedure. Even when catheter ablation alone proved insufficient to achieve complete block due to hindrance by the tricuspid valve patch, surgical ablation utilizing catheter ablation sites as surgical landmarks enabled arrhythmia control.

## Lead author biography


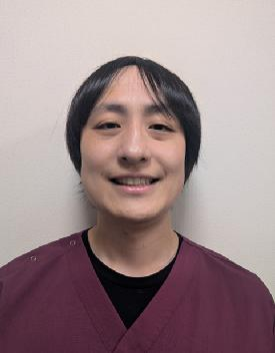


Ken Hayashi, MD, works in the Department of Cardiology at Osaka Women’s and Children’s Hospital, Japan. His clinical and research interests focus on paediatric cardiology, foetal cardiology, and critical care medicine. He is also engaged in medical English scientific writing and manuscript preparation. His experience includes clinical case management, cardiac imaging, and research related to interventional cardiology.

## Supplementary Material

ytag777_Supplementary_Data

## Data Availability

Non-identifiable data related to this case report will be made available upon reasonable request to the corresponding author.
